# Cervical cancer prevention methods: awareness and use among urban Ghanaian women in Cape Coast, West Africa

**DOI:** 10.3332/ecancer.2023.1626

**Published:** 2023-11-13

**Authors:** Abdoul Azize Diallo, Nathaniel Nii A Codjoe, Sebastian Ken-Amoah, Evans Kofi Agbeno

**Affiliations:** Department of Obstetrics and Gynaecology, School of Medical Sciences, University of Cape Coast, Cape Coast, Ghana

**Keywords:** Cape Coast, cervical cancer, awareness, pap smear, HPV vaccination

## Abstract

**Background:**

Cervical cancer is the fourth most frequent malignancy and common cause of mortality in women worldwide, despite being one of the most preventable female cancers.

**Objectives:**

The aim of the study was to assess the awareness and knowledge of patients about cervical cancer prevention methods and the use of these methods by women in an urban setting.

**Method:**

A cross-sectional study design was employed. A census was conducted to include all women (*n* = 153) who met the inclusion criteria and attended the gynaecology clinic of the Cape Coast Teaching Hospital from May to July 2022 for various gynaecological reasons. Data were collected using a structured questionnaire adapted from the Cervical Cancer Knowledge Prevention-64.

**Results:**

The mean age was 40.0 years and ranges between 18 and 78 years. The majority of study participants had at least a secondary school level of education (78.8%), and almost all had at least a primary school education (95.4%). Most of the respondents (64.7%) were not aware of cervical cancer. Among those who had awareness, 64.8% of them knew about the existence of prevention methods; pap smear was the most common known method of prevention. There was a statistically significant association between the respondent’s educational level and knowledge of the existence of cervical cancer prevention methods and the usage of pap smear. Only 16.3% of our study population has ever used a preventive method.

**Conclusion:**

More than half of the participants were not aware of cervical cancer and its preventive methods, and those who were aware had insufficient knowledge, which translated to very low usage of cervical cancer preventive methods. There is an urgent need to intensify public education on cervical cancer.

## Introduction

Cervical cancer is the fourth most frequent malignancy in women worldwide, and it also ranks as the fourth most common cause of mortality in women [1]. Cervical cancer is a major cause of morbidity and mortality in lower-middle-income countries [2]. In 2018, 51% of the new cervical cancer cases worldwide were reported to have occurred in women from lower-middle-income countries [3]. In Ghana, it is the second-commonest cancer affecting women [4].

In 2020, it is estimated that about 604,000 new cases and 342,000 deaths from the disease were recorded [5]. Due to the high mortality associated with cervical cancer, preventive methods are pivotal in reducing the disease burden. There are two main forms of cervical cancer prevention methods: primary prevention and secondary prevention [6]. Primary prevention thus mainly involves Human papillomavirus (HPV) vaccination [7]. Other primary prevention methods include delaying sexual debut, increasing condom use, and having fewer lifetime sexual partners [8]. Secondary prevention involves screening and management of premalignant lesions. Screening involves cytological examination using a Papanicolaou (pap) smear, liquid-based cytology, and visual inspection with acetic acid (VIA) [2]. Other screening methods include HPV-DNA testing.

Despite the availability of effective prevention methods across the country, the number of cases of cervical cancer continues its upward trajectory, likely due to low usage of the prevention services [3]. There is a certain amount of available literature in relation to cervical cancer prevention awareness in Ghana: two of the studies are qualitative exploratory [9, 10] and the quantitative ones are household surveys, which focused only on pap smear as a preventive method and targeted rural and semi-rural populations in limited geographical locations [11, 12]. In view of that, our study, which is quantitative and hospital-based, and targeted urban women with heterogenicity in socio-demographic features and adopted the cervical cancer knowledge prevention-64 (CCKP-64) questionnaire [13] was carried out to assess women’s awareness and their usage of cervical cancer primary as well as secondary preventive methods.

## Methodology

### Study design

A hospital-based cross-sectional study design was conducted over a period of 3 months (from May to July 2022) in Cape Coast, precisely at the Cape Coast Teaching Hospital. This study design allowed for exposures and outcomes relevant to the study to be examined at the same time, and it was relatively affordable. This was the first type to be conducted in our setting; the expectation was to have a snapshot of the situation, which could pave the way for cohort or other study designs.

### Population

According to the 2021 Population and Housing Census, the Cape Coast Metropolis has a population of about 189,924, with about 97,135 adult females. During the study period, 914 women attended the gynaecology clinic of the Cape Coast Teaching Hospital.

All women who visited the gynaecological clinic at Cape Coast Teaching Hospital during the study period constituted the study population.

### Inclusion criteria

- All women over 18 years who attended the gynaecological clinic at Cape Coast Teaching Hospital.

### Exclusion criteria

- Women who presented in an emergency situation.
- All women who declined to participate in the study.

### Sampling technique and sample size

A random sampling was performed on each Outpatient department (OPD) day (Mondays–Fridays) during the study period to select between 4 and 6 patients per day. Each day, all the patients booked for consultation on that specific day were listed and then the respondents were selected randomly from the list.

To estimate the sample size, the Cochran formula applicable for cross-sectional studies was used [14].

- *N*˳= *Z*^2^*P*(1−*P*)/*e*^2^
- *N*˳ is the sample size;
- *Z*-value is found in a *Z* table (1.96 for a confidence interval of 95%);
- *P* is the estimated proportion of the population that has the attribute in question (0.5); and
- *e* is the margin of error which is set at 0.05.

Based on a previous population study in Ghana, the prevalence of women who have knowledge about the prevention of cervical cancer was 8% [11]. In applying the formula and using the proportions *p* = 8% and 1-*P* = 92%, a minimum sample size of *N* = 113 was calculated.

### Data collection instrument

A structured, pre-designed and pretested questionnaire adapted from the CCKP-64 questionnaire was used in data collection [13]. The questionnaire assessed the socio-demographics of the participants, their general knowledge about cervical cancer, and their knowledge about preventive methods.

### Data collection procedure

The respondents were recruited from Mondays to Fridays during the study period at the Gynaecology OPD of the Cape Coast Teaching Hospital, and the purpose and content of the research were explained to them, and their written or thumbprint consent was obtained. Afterward, the respondents were taken to a designated consulting room, so they would be more comfortable responding to the questionnaire.

The questionnaire was an interviewer-administered questionnaire by one of the investigators (second author), who can speak fluently in the local dialects and understand the cultural context to be able to ask the relevant questions in an understandable manner for the respondents. Each interview lasted between 25 and 30 minutes. Also, for the patients who were unable to read, the contents of the consent form were read out to them.

### Data analysis

Analysis was done using the Statistical Package for Social Sciences (SPSS version 26.0) and Microsoft Excel 2016. The association between variables was tested using the Fisher’s exact test. It was reported using a *p*-value with a significance level of 0.05.

### Reliability

The items of this survey questionnaire were extracted from the CCKP-64 questionnaire [13] which has a Cronbach alpha coefficient of 0.71 for the whole questionnaire, and specifically, sections of the questionnaire were as follows: 0.06 for general knowledge about the disease; 0.81 for assessment of risk factors; 0.69 for knowledge about primary prevention; and 0.70 for secondary prevention.

### Validity

The validity of our data was improved by the use of the structured, pre-designed questionnaire adapted from the CCKP-64, which has been tried, tested, and used in several similar studies. Sufficient time was allocated to each participant, and the questionnaires were administered by the research team member.

### Ethical considerations

Ethical clearance was obtained from the Cape Coast Teaching Hospital Ethical Review Committee (CCTH ERC/EC/2021/087) and the University of Cape Coast Institutional Review Board (UCCIRB/CHAS/2022/01). Informed consent was obtained from all study participants. It was ensured that there was confidentiality and anonymity throughout data collection and management by ensuring that participants were identified by serial numbers and not by name.

## Results

### Socio-demographics

The total number of surveyed participants was 153, with a response rate of 100% (153/153). The mean age was 40.0 years with an SD of 14.9 and ranges between 18 and 78 years. Most of the respondents (69.0%) were 30 years or older. The majority of the respondents (64.7%) were workers, and Christianity was the most practiced religion (83.7%). About 50.0% of the participants were married. The majority of study participants had at least a secondary school level (78.8%). The results are summarised below in Table 1.

### Awareness and general knowledge about cervical cancer

Only 35% (54/153) of the women interviewed were aware of cervical cancer. Among those who were aware of cervical cancer, 63.0% of them knew it can lead to a terminal illness, 52.0% knew cervical cancer is caused by an infective agent and 64.8% knew about the existence of preventive methods. The findings on awareness and general knowledge of cervical cancer are summarised in Table 2.

### Knowledge about the existence and types of cervical cancer prevention methods

Among the 35 (22.8%) respondents of the study population who knew about the existence of preventive methods against cervical cancer, the pap smear was the most commonly known (85.7%), while only a few of them (20.0%) knew about the existence of HPV vaccines. Table 3 summarises the findings obtained from the study.

### Knowledge and usage of pap smear

About 85.7% (30/35) of the women among those who know that cervical cancer can be prevented, knew of pap smear as a screening method of cervical cancer. Out of this number, 25 respondents (83.0%) had done the test at least once before.

The remaining five respondents who had never done the test despite knowing it, mentioned fear of pain and discomfort as a reason.

### Association between socio-demographics and awareness on the existence of cervical cancer prevention methods

The Fisher exact test, with a significance level of 0.05 (*p*-value), was conducted to determine the association between the respondents’ age, educational level and marital status with regard to knowledge of the existence of cervical cancer prevention methods. The respondent’s educational level was statistically associated with knowledge of the existence of cervical cancer prevention methods; however, age and marital status were not significantly associated. Table 4 summarises the association between socio-demographic features and knowledge of the existence of cervical cancer prevention methods.

### Association between socio-demographics and usage of pap smear

Using the Fisher exact test with a significance level of 0.05 (*p*-value), it was found that the respondents’ age, level of education and marital status were statistically associated with the usage of pap smear. Table 5 summarises the association between socio-demographic features and the use of pap smear.

## Discussion

### Socio-demographics

Most of the respondents were towards the upper limit of their reproductive ages, with a mean age of 40.0 years. This can be explained by the fact that the recruitment of the respondents was done at the general gynaecology clinic, which excludes obstetrics and fertility consultations. Otherwise, the younger patients would have been the majority. Most of our subjects have at least secondary school education and almost all have attained primary school level, which puts them in the category of educated.

The age distribution in this study is similar to a study in India that also reported a mean age of 41.8 years [8]. Other similar studies conducted in Tanzania and Ethiopia reported a mean age of 32.86 and 35.7 years, respectively [11, 10], which are younger than our population.

### Awareness and general knowledge about cervical cancer and its preventive methods

Most of the participants (64.7%) did not have any awareness or knowledge about cervical cancer preventive methods. This finding was surprising because of the level of education of the participants (most having at least secondary school education) and the statistically significant association between educational level and knowledge of prevention methods. That is, it was expected of them to have a certain awareness. It is worth mentioning that, in the country, there is no specific national program for education or prevention of cervical cancer, even though the Ghana Health Service is running other ten prevention-related programs [15], which limit the availability of sources of knowledge. The results correlate with several studies carried out on awareness and knowledge of cervical cancer amongst women, which showed a lack of awareness and inadequacy of knowledge on cervical cancer in Sub-Saharan Africa [2, 6, 16]

Only 52% of those with awareness of cervical cancer knew that an infective agent was the root cause. This shows that even for those with awareness of cervical cancer, their knowledge was found to be limited. This is similar to studies carried out in Kenya and Tanzania, which showed that more than half of the participants did not know enough about it [6, 16].

### Knowledge about cervical cancer prevention methods

Among the few respondents who knew about cervical cancer, most of them had knowledge of at least one preventive method, and pap smear was the most known method. This could be explained by the fact that pap smear services are the most widely available and affordable cervical cancer preventive method in urban Ghana as well as other West African countries [10, 16]. These findings contrast with other studies, which showed that most women did not know about the availability of avenues for the prevention of cervical cancer [6, 11].

### Usage of pap smear

It was found that only 16.3% of our study population (*n* = 25) had done the pap smear test at least once before. The low uptake of pap smear observed is in line with a study in the general population at Elmina, which showed only 3.3% of respondents had had a pap smear test [6]. Possible reasons for this low coverage could be the lack of or inadequacy of public education on cervical cancer itself and the various prevention methods available. Another possible explanation could be concern about pain and discomfort, as testified by five respondents in the current survey and also found in other studies [17, 18]. This reveals the need to integrate women’s fears and misconceptions when designing cervical cancer awareness programmes.

### Association between socio-demographics and usage of pap smear

The respondents’ age, educational level and marital status were statistically associated with usage of pap smears. Similar findings were made in Taiwan [19], where age is strongly associated, particularly for women below the age of 30 and over the age of 65, as well as marital status and the level of education. A study about the uptake of pap tests In Ghana [20], it was also found that the educational level was associated but not the other socio-demographic features. This emphasises the need to educate women alongside other interventions in the fight against cervical cancer.

## Conclusion

More than half of the participants were not aware of cervical cancer and its preventive methods, and those who were aware had insufficient knowledge, which translated to very low usage of cervical cancer preventive methods. Also, there was a statistically significant association between both the respondent’s educational level and knowledge of the existence of cervical cancer prevention methods, as well as the usage of pap smear. There is an urgent need to intensify public education on cervical cancer in designing awareness programmes that should take into account misconceptions and fears about screening and also increase women’s level of education.

## Author contributions

The conception of the study and supervision was done by Diallo. Codjoe Nii did the literature review, collected the data and did the analysis and discussion, Ken-Amoah did the manuscript write up, and Agbeno did the proof reading of the manuscript.

## Conflicts of interest

None.

## Funding

No funding was provided for this project.

## Figures and Tables

**Table 1.** Socio-demographics of study participants (*N* = 153).

Variable		Frequency	Percentage
Age	18–29	47	30.7
	30–44	51	33.3
	45–59	35	22.9
	>60	20	13.1
Religion	Christian	128	83.7
	Muslim	25	16.3
Marital status	Married	77	50.3
	Single	52	34.0
	Cohabitation	24	15.7
Highest educational level	No education	7	4.6
	Primary	27	17.6
	JHS	38	24.8
	SHS	37	24.2
	Tertiary	44	28.8
Occupation	Farmer	28	18.3
	Government employed	28	18.3
	Self employed	5	3.3
	Trading	38	24.8
	Student	41	26.8
	Unemployed	13	8.5

**Table 2.** Awareness and general knowledge about cervical cancer (*N* = 153).

Variable		Frequency	Percentage
Awareness about Ca cervix? n = 153	No	99	64.7
	Yes	54	35.3
Can it be a terminal illness? *n* = 54	Do not know	18	33.3
	No	2	3.7
	Yes	34	63.0
Is cervical cancer caused by an infection? *n* = 54	Do not know	26	48.1
	Yes	28	51.9
Knowledge on the existence of preventive methods? *n* = 54	Do not know	18	33.3
	No	1	1.9
	Yes	35	64.8

**Table 3.** Table showing which prevention methods participants know about the existence (*n* = 35).

Known type of method of prevention	Frequency	Percentage (%)
Pap smear	30	85.7
Vaccination	7	20.0
HPV DNA testing	5	14.3
VIA	5	14.3.

**Table 4.** Association between the socio-demographic features and awareness of cervical cancer prevention methods.

Characteristics	Had awareness on prevention methods	Had no awareness on prevention methods	Total	*p*-values
Age				
<30 years	18 (11.7%)	29 (18.9%)	47 (30.7%)	0.71
≥30 years	36 (23.5%)	70 (45.7%)	106 (69.2%)	
Highest educational level				
Secondary and tertiary	49 (32.0%)	70 (45.7%)	119 (77.7%)	0.004
Primary + no education	5 (3.2%)	29 (18.9%)	34 (22.2%)	
Marital status				
Married and cohabiting	38 (24.8%)	63 (41.1%)	101 (66.0%)	0.47
Singles	16 (10.4%)	36 (23.5%)	52 (33.4%)	

**Table 5.** Association between the socio-demographic features and ever used pap smear as preventive methods.

Characteristics	Ever used pap smear	Never used pap smear	Total	*p*-values
Age				
<30 years	3 (%)	44 (%)	47 (30.7%)	0.01
≥30 years	22 (%)	84 (%)	106 (69.2%)	
Highest educational level				
Secondary and tertiary	23 (%)	96 (45.7%)	119 (77.7%)	0.04
Primary + no education	2 (%)	32 (18.9%)	34 (22.2%)	
Marital status				
Married and cohabiting	21 (%)	80 (41.1%)	101(66.0%)	0.02
Singles	4 (%)	48 (23.5%)	52 (33.4%)	

## Questionnaire

Socio-demographics

1. Age (years)
  …………………
2. Religion
  - No religion
  - Christian
  - Muslim
  - Traditional
  - Other
3. Tribe
  - Asante
  - Fante
  - Ga/Adangbe
  - Ewe
  - Akyem
  - Other
4. Highest level of education
  - None
  - Primary
  - JHS
  - SHS
  - Tertiary
5. Occupation
  - Unemployed
  - Trading/Vending
  - Farmer
  - Government employed
  - Self-employed
  - Student
6. Marital status
  - Single
  - Married
  - Co-habitation
  General knowledge about cervical cancer
7. Have you ever heard about cervical cancer?
  - Yes
  - No
    *If response is ‘no’, skip to 16*
8. If yes to 7, what have you heard?
  **………………………………………………………………………………………………………………………………………..**
9. Can cervical cancer be a terminal illness?
  - Yes
  - No
  - Do not know
10. Is cervical cancer caused by an infection?
  - Yes
  - No
  - Do not know
11. Is there an effective method that significantly reduces the risk of this disease?
  - Yes
  - No
  - Do not know
12. Do you know anyone who had cervical cancer?
  - Yes
  - No
  - Do not know
13. If yes to 13, who was it?
  - Family
  - Friend
  - Other
14. Do you think this disease could affect you in the future?
  - Yes
  - No
  - Do not know
15. If yes to 14, how?
  **……………………………………………………………………………………………………………………..**
  Knowledge about primary prevention (vaccination)
16. Have you heard about the vaccine against cervical cancer?
  - Yes
  - No
    *If response to 16 is ‘no’, skip to 22*
17. If yes to 16, is it available in Ghana?
  - Yes
  - No
  - Do not know
18. If yes to 17, is it free?
  - Yes
  - No
  - Do not know
19. Do you think it is effective?
  - Yes
  - No
  - Do not know
20. Do you know where you can get vaccinated?
  - Yes
  - No
21. If yes to 20, where specifically?
  **………………………………………………………………………………………………………………………….**
22. Have you ever been vaccinated against cervical cancer?
  - Yes
  - No
    *If response is ‘no’, skip to 24*
23. If yes to 22, did you get any adverse effects?
  - Yes
  - No
24. If no to 22, do you have plans of getting vaccinated?
  - Yes
  - No
25. Will you recommend use of vaccine to other people?
  - Yes
  - No
  Knowledge about secondary prevention (screening)
  A. Cytological examination
26. Have you ever heard about cytological examination (Pap smear or liquid-based cytology)?
  - Yes
  - No
  *If answer to 26 is ‘no’, skip to 36*
27. Is it a test that gives a 100% chance of early diagnosis of premalignant lesion of the cervix?
  - Yes
  - No
  - Do not know
28. Is the test painful?
  - Yes
  - No
29. Is it a time-consuming test?
  - Yes
  - No
30. Is it possible to be tested free of charge?
  - Yes
  - No
  - Do not know
31. Is it sufficient to do the test only once in order to eliminate the risk of cervical cancer?
  - Yes
  - No
  - Do not know
32. Can the test cause serious complication?
  - Yes
  - No
  - Do not know
33. Is it possible for the Pap smear to increase the susceptibility to cervical cancer in the future?
  - Yes
  - No
  - Do not know
34. Do you think you should undergo cytological examination?
  - Yes
  - No
  - Do not know
35. Have you ever been screened with cervical cytology ?
  - Yes
  - No
  B. VIA
36. Have you ever heard about VIA?
  - Yes
  - No
37. Is it a test that gives a 100% chance of early diagnosis of cervical cancer?
  - Yes
  - No
  - Do not know
38. Is the test painful?
  - Yes
  - No
39. Is it a time-consuming test?
  - Yes
  - No
40. Is it possible to be tested free of charge?
  - Yes
  - No
  - Do not know
41. Is it sufficient to do the test only once in order to eliminate the risk of cervical cancer?
  - Yes
  - No
  - Do not know
42. Can the test cause serious complication?
  - Yes
  - No
  - Do not know
43. Is it possible for the VIA to increase the susceptibility to cervical cancer in the future?
  - Yes
  - No
  - Do not know
44. Do you think you should undergo the test?
  - Yes
  - No
  - Do not know
